# Understanding iron homeostasis in MDS: the role of erythroferrone

**DOI:** 10.3389/fonc.2024.1404817

**Published:** 2024-05-21

**Authors:** Mohammed L. Abba, Vladimir Riabov, Daniel Nowak, Wolf-Karsten Hofmann, Tobias Boch

**Affiliations:** ^1^ Department of Hematology and Oncology, Medical Faculty Mannheim, Heidelberg University, Mannheim, Germany; ^2^ Department of Hematology and Oncology, University Hospital Mannheim, Mannheim, Germany

**Keywords:** myelodysplastic neoplasms, erythroferrone, iron overload, SF3B1 mutations, hepcidin

## Abstract

Myelodysplastic neoplasms (MDS) are a heterogenous group of clonal stem cell disorders characterized by dysplasia and cytopenia in one or more cell lineages. Anemia is a very common symptom that is often treated with blood transfusions and/or erythropoiesis stimulating factors. Iron overload results from a combination of these factors together with the disease-associated ineffective erythropoiesis, that is seen especially in MDS cases with SF3B1 mutations. A growing body of research has shown that erythroferrone is an important regulator of hepcidin, the master regulator of systemic iron homeostasis. Consequently, it is of interest to understand how this molecule contributes to regulating the iron balance in MDS patients. This short review evaluates our current understanding of erythroferrone in general, but more specifically in MDS and seeks to place in context how the current knowledge could be utilized for prognostication and therapy.

## Introduction

A daily production of more than 200 billion new erythrocytes is needed to maintain our red blood cell count (1). This massive cell output is dependent on sufficient iron supply, making erythropoiesis the predominant consumer of iron. This system is challenged, particularly in situations of acute blood loss such as hemorrhage or hemolysis, when the oxygen-carrying capacity of red cells is compromised. Despite fluctuating changes in red blood cell production and availability of dietary iron, plasma iron levels remain stable indicating a tightly regulated iron-homeostatic system. The main components of this system are the cellular iron exporter ferroportin and the peptide hormone hepcidin, which is able to block ferroportin (2). Hepcidin is primarily produced by hepatocytes and is a negative regulator of iron uptake into the blood stream. Increased hepcidin levels block intestinal iron absorption and macrophage iron recycling from aged or defective erythrocytes. Suppression of hepcidin can increase iron absorption and mobilization from stores, mainly to support expanding erythropoiesis under anemic conditions. This suppression of hepcidin was shown to require erythropoietic activity (3). Treatment with erythropoiesis stimulating factors (ESF) such as erythropoietin and conditions that further trigger erythropoiesis (e.g. hypoxia, hemolysis, excessive bleeding) repress hepcidin expression in mice (4). Similarly, in humans, the administration of erythropoietin resulted in a prompt decrease of hepcidin (5). In 2014, significant insight was gained into how erythropoiesis regulated iron supply to guarantee the appropriate delivery of iron to proliferating erythroid progenitors (6). Ganz et al. identified erythroferrone (ERFE) as a new erythroid regulator responsible for the early suppression of hepcidin levels after erythropoietic stimulation. ERFE is produced almost exclusively by erythroblasts within the bone marrow and can induce hepcidin suppression by inhibiting hepatic BMP/SMAD signaling (7). ERFE-deficient mice are phenotypically normal under unstressed conditions. However, after hemorrhage, these mice fail to suppress hepcidin and experience a delay in recovery from blood loss (8). More recently, Coffey et al. generated transgenic mouse models of graded erythroid ERFE overexpression that resulted in ERFE dose-dependent iron overload. Intriguingly, the mice showed developmental abnormalities at high ERFE overexpression levels, most likely due to impaired organ-specific BMP signaling (9). Besides its obvious role as a physiological erythropoietic regulator of iron metabolism, ERFE was also identified as a pathological suppressor of hepcidin, for example in a Hbb^th3/+^ mouse model of non-transfusion dependent thalassemia intermedia, where it contributed to the pathological iron overload associated with this disease (10). After mating Hbb^th3/+-^ thalassemia mice with ERFE-deficient mice, only a moderate improvement in iron overload was observed. Although ERFE ablation completely reversed the hepcidin mRNA suppression, the development of iron overload was only partially averted with no effect on anemia. Undoubtedly, this indicated that other factors besides the ERFE/hepcidin axis contribute to the pathological iron overload in thalassemia.

In addition to its physiological role in iron homeostasis and in acute conditions such as blood loss, the ERFE/hepcidin axis is also chronically deregulated in myelodysplastic neoplasms (MDS).

## Iron homeostasis in MDS

Myelodysplastic neoplasms (MDS) are malignant clonal diseases of hematopoietic stem cells (HSCs) exemplified by single or multilineage dysplasia, ineffective hematopoiesis and enhanced risk of transformation into acute myeloid leukemia (AML). Iron overload manifests in MDS patients prior to transfusion dependency because ineffective erythropoiesis suppresses hepcidin production in the liver inducing a massively enhanced intestinal iron uptake. Subsequently, the inability of erythroid progenitors to differentiate into mature red blood cells precipitates an increased demand that funnels an increased iron consumption and iron overload (11). Santini et al. made use of an improved mass-spectrometry based method to analyze serum hepcidin levels in patients with different subtypes of MDS (12). The lowest hepcidin levels were recorded in refractory anemia with ring sideroblasts and the highest in refractory anemia with excess blasts or in chronic myelomonocytic leukemia. In multivariate analyses, MDS subtypes remained significant predictors of hepcidin levels (12). The inappropriately low serum hepcidin levels in MDS subtypes with ring sideroblasts were later attributed to coexisting SF3B1 mutations (13).

ERFE’s role as the principal erythroid regulator of iron homeostasis due to its effect on the hepcidin/ferroportin axis in humans is already well studied, just as its pathological role in anemias with ineffective erythropoiesis, for example in thalassemia intermedia, which was demonstrated in *in-vivo* models. However, the significance of ERFE in the context of dysplastic erythropoiesis still remains rather elusive. This has changed recently, with new information coming to light and therefore, in this short review we aim to highlight recent important findings concerning the role of ERFE in MDS and discuss future perspectives.

## Increased levels of ERFE in MDS with ring sideroblasts

The subtype of MDS with ring sideroblasts shows markedly reduced levels of hepcidin (12). Due to the aberrant accumulation of intracellular iron into mitochondria, it is assumed that there is a lack of intracellular iron availability for proper heme synthesis (14). Sideroblasts are therefore iron overloaded but falsely appear to the organism as ineffective erythroid progenitors, which are iron deficient. A consequence of this perceived “pseudo”-iron deficiency is the ERFE driven suppression of hepcidin and subsequent increased iron uptake through the intestinal system. In a study by Miura et al. the expression level of ERFE in MDS was further stratified according to WHO subtypes (15). ERFE expression was increased in all MDS subtypes compared to healthy controls, but was specifically pronounced in cases with ring sideroblasts (MDS-RS-SLD and MDS-RS-MLD). This data is in line with the observation that MDS-RS patients already present with elevated levels of ferritin before they become transfusion dependent (16).

## A variant of erythroferrone disrupts iron homeostasis in SF3B1-mutated MDS

In approximately 90% of MDS with ring sideroblasts the splicing factor gene SF3B1 is mutated. In the landmark study by Malcovati et al., the mutated SF3B1 gene had a positive predictive value for ring sideroblasts of 98% (17). Mutations in SF3B1 can be detected at the stem cell level, indicating a driving mechanism for the dysplastic phenotype (18, 19). SF3B1 mutations result in aberrant splicing, mostly by mis-selection of alternative branch sites that can enable otherwise cryptic 3`splice sites. The aberrant transcripts are mostly subjected to nonsense-mediated decay (NMD) due to premature termination codons. However, some transcripts are NMD insensitive and become translated into functionally altered proteins. How this missplicing can ultimately lead to disease phenotypes is not yet fully understood and still largely elusive. Recently, Bondu et al. provided important data to better link splicing defects with iron overload in myelodysplasia (20). They performed gene expression analyses from bone marrow mononuclear cells of MDS patients comparing n=21 samples with SF3B1^mut^ to n= 6 samples with SF3B1^wt^ as well as healthy controls.

They identified ERFE as one of the genes with increased expression in SF3B1^mut^ MDS. They also detected differentially expressed 5` and 3` splicing junctions that allowed for distinction between SF3B1^mut^ and SF3B1^wt^. The alternative junctions of the ERFE gene could be attributed to a cryptic 3`splicing site between exons 2 and 3 that did not result in premature termination, but in an aberrant transcript, which was enlarged by 12 additional nucleotides in the open reading frame. This newly detected enlarged ERFE variant, “ERFE^VPFQ^”, could be detected in all MDS SF3B1^mut^ samples and constituted around 25% of total ERFE transcripts. In MDS lacking SF3B1 mutations this ERFE variant represented only 0.2%, which indicated almost exclusivity of the new variant for mutated SF3B1. In primary samples of MDS with mutations in other epigenetically relevant genes such as SRSF2, U2AF1 or ZRSR2, the ERFE^VPFQ^ transcript could not be detected. In one MDS patient sample with the very interesting constellation of one SF3B1 allele being mutated and the other one being deleted, ERFE^VPFQ^ was the dominantly expressed transcript. Additionally, since the ERFE^VPFQ^ transcript was missing in patient samples of MDS-RS with SF3B1^wt^ TET2^mut^ SRSF2^mut^, congenital sideroblastic anemia with ALAS2^mut/del^ and ß-thalassemia, it is very likely that ERFE^VPFQ^ expression is independent of the amplified erythroid bone marrow compartment and the presence of ring sideroblasts.

The aberrant ERFE transcript is translated into a protein with a four amino acid in-frame insertion at position 108 (valine-proline-phenylalanine-glutamine, VPFQ). ERFE^VPFQ^ could be identified in cell lysates of SF3B1^mut^ erythroblasts. Both, ERFE^wt^ and ERFE^VPFQ^ were able to reduce hepcidin production in hepatocellular carcinoma cell lines. Bondu et al. made use of an immunoassay for human serum ERFE developed by the Ganz lab (21) to measure total serum ERFE levels in their MDS cohort as well as in healthy individuals (20). In that study, total serum ERFE levels were significantly higher in MDS patients and particularly in cases with SF3B1^mut^, where the increased serum ERFE levels included higher expression of the ERFE^VPFQ^ variant (135.0 ± 72.5 ng/ml in the variant compared to 62.1 ± 36.7 ng/ml in the wild type). ERFE levels as well as hepcidin/ferritin ratios in different MDS subtypes are also provided in that study.

In cases of SF3B1^mut^ MDS there was a significant (two-fold) increase in ferritin levels and a corresponding decrease of hepcidin concentration compared to SF3B1^wt^ MDS leading to a markedly lower hepcidin/ferritin ratio. In early stage MDS, when patients have not been regularly transfused, ERFE as well as hepcidin and mutated SF3B1 are independent predictors of hyperferritinaemia (20). Since both, the SF3B1^mut^ and SF3B1^wt^ MDS patients had a similar red blood cell transfusion burden, it is likely that the increase in ERFE is independent from the transfusion frequency.

Despite a generally increased serum EPO concentration in MDS, there was no correlation with ERFE concentrations, neither in MDS with SF3B1^mut^ nor in the SF3B1^wt^ setting. This is a particularly interesting observation since data from studies in murine models clearly indicated a stimulation of ERFE transcription upon stimulation with EPO, that mainly occurs in erythroblasts (6). Myelodysplastic erythropoiesis seems to lack this EPO driven stimulation of ERFE secretion.

**Figure 1:**
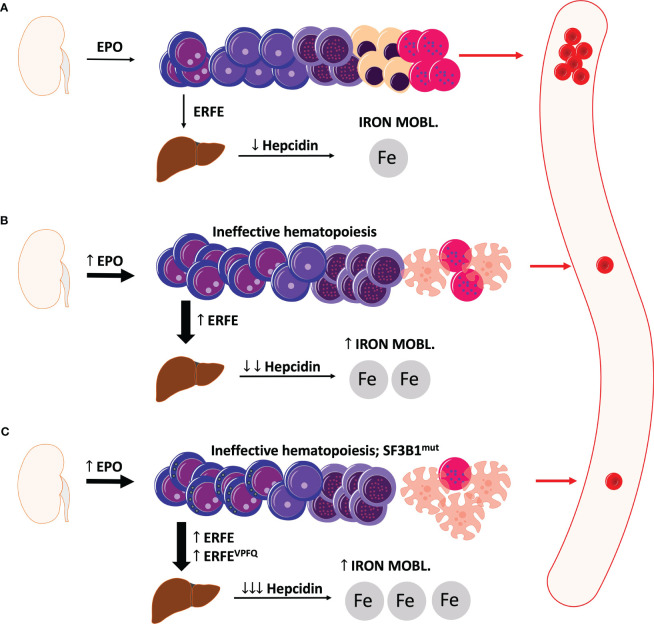
Schematic illustration of erythropoiesis and erythroferrone in health and MDS. **(A)** Under physiologic conditions, sufficient levels of erythroferrone are produced to regulate the production of hepcidin. Significant levels of hepcidin entail an optimal uptake of dietary iron for physiological erythropoiesis. **(B)** In MDS, ineffective erythropoiesis resulting from apoptosis of late-stage precursors results in anemia with an increased production of EPO and also erythroferrone. Elevated levels of erythroferrone significantly repress hepcidin production leading to enhanced iron mobilization and absorption. **(C)** In MDS with ring sideroblasts and SF3B1 mutations, an additional erythroferrone variant further aggravates iron uptake leading to considerable iron overload. ERFE, erythroferrone. The depicted cells show progressive cell differentiation/maturation in the bone marrow (starting from left to right): proerythroblasts, basophilic erythroblasts, polychromatic erythroblasts, normoblasts, reticulocytes, and erythrocytes. Apoptotic cells are depicted as cells with the distorted morphology and blebbing.

**Figure 1** displays a schematic illustration of erythropoiesis and effects of ERFE in health and MDS with and without SF3B1^mut^.

## High ERFE expression in CD71^+^ erythroid progenitors predict superior survival in MDS

When Bondu and colleagues differentiated CD34^+^ precursors from MDS patients with both SF3B1^mut^ and SF3B1^wt^, they also detected an erythroid lineage-restricted expression of ERFE, including the variant ERFE^VPFQ^ (20). This lineage restriction of ERFE expression was also confirmed by our group, where we showed that ERFE was exclusively and highly overexpressed in CD71^+^ erythroid precursors within bone marrow mononuclear cells of MDS patients (22).

The analysis of ERFE expression in sorted subpopulations of hematopoiesis showed an interindividual (quantitative) variance, both in MDS and in healthy controls. We further analyzed ERFE expression in CD71^+^ cells of a larger cohort of MDS patients (n=111) and healthy controls (n=52), where it was shown that ERFE expression was significantly upregulated in low/int-1 MDS, which corresponds to the increased ERFE expression in MDS as shown by Miura et al. (15). Again, expression levels in MDS showed a strong heterogeneity and ERFE expression was higher in SF3B1^mut^ MDS compared to SF3B1^wt^. We further correlated our expression data with survival data. High ERFE expression was associated with significantly better survival probability expressed by a hazard ratio of 0.35. The effect was further emphasized in the low risk/int-1 sub-group with a hazard ratio of 0.12. This survival benefit in low-risk MDS might have been attributed to increased SF3B1 mutations since it is well-known that SF3B1 mutations are beneficial from a prognostic point of view (23, 24). Therefore, in an important subanalysis, the authors showed that high EFRE expression did not just mirror SF3B1 mutational status but was also retained in the SF3B1^wt^ cohort. In multivariate Cox regression analysis increased ERFE expression appeared as an independent predictor of survival.

The SF3B1^mut^ CD71^+^ erythroid progenitors also expressed the ERFE^VPFQ^ variant with a median fraction of around 40%. An elevated ERFE^variant^/ERFE^total^ ratio only showed a trend toward better survival, but without statistical significance.

## Discussion

Presently, many aspects relating to the biology of ERFE in MDS still remain elusive and the biology of ERFE in MDS is still poorly defined. Whereas its role in iron homeostasis as a physiological erythropoietic regulator of iron metabolism is rather well-established, the involvement of ERFE in regulation of iron metabolism in MDS and also its contribution to the pathogenesis of myelodysplasia is de facto still unknown. In contrast to preclinical *in-vitro* and *in-vivo* studies in other forms of anemia with defective erythropoiesis, mainly thalassemia, there have not been any *in-vivo* studies in MDS models so far. *In-vivo* studies of MDS have been hampered by the lack of genetic mouse models or robust xenografts. In the meantime, some valid xenograft approaches have been published that allow e.g. treatment with erythropoiesis or thrombopoiesis stimulating agents (25). Therefore, it would be worthwhile to treat low risk MDS xenografts with either ERFE agonists or antagonists. If ERFE expression in erythropoietic progenitors translated into a survival benefit, could this effect be abrogated by ERFE inhibition and also the other way around? Can the inferior survival of “ERFE low” expressing MDS be improved by substitution of exogenous ERFE? *In-vivo* analyses of ERFE treated xenografts would also allow for gene expression profiling of different hematopoietic subgroups and components of the hematopoietic niche upon ERFE treatment.

Another aspect of ERFE that has not been addressed so far, is the question if, and to which extent ERFE exerts local effects within the bone marrow of MDS patients and also in healthy individuals. Myonectin, a myokine secreted by skeletal muscle cells in order to effect systemic lipid homeostasis, was shown to be identical to ERFE (26, 27). ERFE/myonectin exerts protective effects on myocardial muscle cells after ischemia-reperfusion injury (24). Therefore, it might be possible that ERFE exerts similar metabolic effects within the bone marrow niche of MDS that could be protective for the residual erythropoiesis and ultimately improve survival probability (22).

Besides these considerations, ERFE expression might also just be an indicator of potent and functional erythropoiesis. In this case ERFE itself might not necessarily have to exert direct effects within the hematopoietic niche. In MDS cases with high ERFE expression the survival benefit might result from the fact that the damage in erythropoiesis is still limited enough to allow erythroid progenitors to better adapt to an anemic state, e.g. by upregulating ERFE. One might assume that MDS patients with higher erythroid ERFE expression might also better respond to initial treatment with erythropoiesis stimulating agents. In our MDS cohort, we did not see any significant association between ERFE low or high status and MDS specific prior treatment including erythropoiesis stimulating agents (22). Risk stratification is particularly important in MDS, both from a prognostic point of view as well as for therapeutic guidance. Scoring systems such as the IPSS classification have been widely implemented (28, 29). ERFE expression was shown to also have prognostic power, which was furthermore independent from SF3B1 mutational status and could successfully stratify patients with normal karyotype into superior and inferior survival (22).

The concept of ERFE as a therapeutic target in MDS currently appears rather controversial. On first thought, it seems most plausible to antagonize ERFE in the early course of MDS when patients have not yet received RBC transfusions but already present with iron overload that can predispose to consecutive organopathy (e.g. hepatopathy, cardiomyopathy and others). In this context antagonizing ERFE would result in increased hepcidin levels that effectively lower the intestinal iron absorption and help to prevent iron overload and the associated toxicity. This concept has already been demonstrated in other forms of anemia with impaired erythropoiesis such as thalassemia. In the Hbb^th3/+^ mouse model of non-transfusion dependent thalassemia intermedia, bone marrow and splenic ERFE expression was increased and also serum ERFE levels were higher (10). By ablating ERFE, the hepcidin suppression could be overcome. As already mentioned, it has to be noted that in these mice the iron overload as well as the anemia could only be partially improved indicating a more complex regulatory mechanism, most likely including hypoxia mediated factors. The same will most likely be true for MDS. ERFE is surely just one player among others orchestrating the response to deranged iron homeostasis whose inhibition might be compensated by other factors. ERFE expression levels were shown to decrease in patients with β-thalassemia after transfusion and increase upon administration of erythropoietin in geriatric patients (21). In both studies by Bondu et al. (20) and Riabov et al. (22), ERFE expression was not influenced by transfusion status and previous treatment with erythropoiesis stimulating agents. ERFE will not be suitable as an indicator for better treatment response upon the administration of such agents.

The discovery of an ERFE variant that results from missplicing due to the common SF3B1 mutation in MDS is so far one of the major findings in ERFE research in MDS. Due to the connection between the expression of the ERFE variant and SF3B1 mutational status it may be plausible to implement ERFE variant levels as a biomarker for clonal hematopoiesis. Since there is currently only an immunoassay described that detects total ERFE levels, implementing ERFE variant as a biomarker would require a tailored immunoassay to specifically detect the variant protein.

Taken together, ERFE is an important component of the physiological response to iron deficiency. Furthermore, it also exerts a pathological function in anemias with defective erythropoiesis. The recent finding of an ERFE variant in SF3B1 mutated MDS and the observation that ERFE expression might be prognostically relevant, have turned ERFE into a potentially interesting new target in MDS. Research in this field is still in the early stages and it will be very exciting to see where this leads particularly in further unraveling the pathogenesis of myelodysplasia and the identification of additional therapeutic targets.

## Author contributions

MA: Writing – original draft, Writing – review & editing. VR: Writing – review & editing. DN: Writing – review & editing. W-KH: Writing – review & editing. TB: Writing – original draft, Writing – review & editing.
